# Surface Modification of Carbon Nanofibers to Improve Their Biocompatibility in Contact with Osteoblast and Chondrocytes Cell Lines

**DOI:** 10.3390/ma14216370

**Published:** 2021-10-25

**Authors:** Wojciech Smolka, Monika Ptas, Agnieszka Panek, Malgorzata Krok-Borkowicz, Marcel Zambrzycki, Maciej Gubernat, Jaroslaw Markowski, Aneta Fraczek-Szczypta

**Affiliations:** 1 Laryngology Department, School of Medicine in Katowice, Medical University of Silesia in Katowice, Poniatowskiego 15, 40-055 Katowice, Poland; wojciech.smolka@op.pl (W.S.); jmarkowski@sum.edu.pl (J.M.); 2 Faculty of Materials Science and Ceramics, AGH University of Science and Technology, Mickiewicza 30 Av., 30-059 Krakow, Poland; monikaptas98@gmail.com (M.P.); krok@agh.edu.pl (M.K.-B.); zambrzycki@agh.edu.pl (M.Z.); Maciej.Gubernat@agh.edu.pl (M.G.); 3 Institute of Nuclear Physics, Polish Academy of Sciences, ul. Radzikowskiego 152, 31-342 Krakow, Poland; agnieszka.panek@ifj.edu.pl

**Keywords:** electrospun carbon nanofibers (eCNFs), surface modification, microstructure, physicochemical properties, biocompatibility, osteoblast and chondrocytes cell lines

## Abstract

The goal of this study is to investigate the influence of different types of modifiers, such as sodium hyaluronate (NaH), graphene oxide (GO), silica oxycarbide (SiOC) and oxidation process (ox) on physicochemical, morphological, and biological properties of electrospun carbon nanofibers (eCNFs). Scanning electron microscopy, X-ray photoelectron spectroscopy and infrared spectroscopy (FTIR) were used to evaluate the microstructure and chemistry of as-prepared and modified CNFs. The electrical properties of CNFs scaffolds were examined using a four-point probe method to evaluate the influence of modifiers on the volume conductivity and surface resistivity of the obtained samples. The wettability of the surfaces of modified and unmodified CNFs scaffolds was also tested by contact angle measurement. During the in vitro study all samples were put into direct contact with human chondrocyte CHON-001 cells and human osteosarcoma MG-63 cells. Their viability was analysed after 72 h in culture. Moreover, the cell morphology and cell area in contact with CNFs was observed by means of fluorescence microscopy. The obtained results show great potential for the modification of CNFs with polymer, ceramic and carbon modifiers, which do not change the fiber form of the substrate but significantly affect their surface and volume properties. Preliminary biological studies have shown that the type of modification of CNFs affects either the rate of increase in the number of cells or the degree of spreading in relation to the unmodified sample. More hydrophilic and low electrically conductive samples such as CNF_ox and CNF_NaH significantly increase cell proliferation, while other GO and SiOC modified samples have an effect on cell adhesion and thus cell spreading. From the point of view of further research and the possibility of combining the electrical properties of modified CNF scaffolds with electrical stimulation, where these scaffolds would be able to transport electrical signals to cells and thus affect cell adhesion, spreading, and consequently tissue regeneration, samples CNF_GO and CNF_SiOC would be the most desirable.

## 1. Introduction

For many years there has been considerable interest in potential uses of carbon nanomaterials such as nanotubes, graphene, and graphene oxide in various fields, including biological and medical sciences [1,2]. Their interesting electrical, mechanical and biological properties mean that the interest in these materials in the fields of medicine and related sciences has not diminished. The potential for functionalization of these materials by means of functional groups, proteins, growth factors or other biological substances has attracted particular interest. Among the areas of medicine particularly interested in carbon nanoforms is regenerative medicine, especially bone and nervous tissue regeneration, and also, in recent years, cartilage tissue. Regenerative medicine is based on scaffolds, i.e., three-dimensional substrates with appropriate porosity, pore size, permeability, durability or surface modifications that allow for cell adhesion, proliferation or differentiation. The forementioned carbon nanoforms, while interesting with regard to the applications described, do not meet all the requirements for an ideal scaffold for tissue regeneration. There have also been some reports indicating their cytotoxic activity or problems with uncontrolled releases of these materials and accumulation in organs or tissues. An interesting solution that allows the combination of the potential of carbon nanoforms with the biomimetic nature of these materials, especially in terms of substrates for the regeneration of bone and cartilage tissue, is the use of electrospun carbon nanofibers (eCNFs). These eCNFs have been considered for this use due to their attractive features such as mechanical, morphological, chemical, electrical and biological properties [3,4]. As mentioned, eCNFs are an electrically conductive material, and as is known for some types of cells/tissues, the electrical properties play an important role in the regenerative or stimulating processes of cell outgrowth. Therefore, the electrical conductivity of tissue substrates, in addition to the physicochemical, structural and microstructural properties, may play an important role in the processes of cell growth, their proliferation or, as in the case of bone tissue, also mineralization. In bone tissue, the generation of electrical signals is a fairly common phenomenon, caused, for example, by the shear force of collagen and deformation of fluid-filled channels, and in turn these electrical signals can play a key role in osteogenesis [5,6,7]. Therefore, electrical stimulation (ES) in combination with conductive scaffold materials is available as an adjunctive therapy to accelerate bone healing. Some researchers indicate that conductive substrates can adequately mimic a cell niche and transfer signals between cells using electricity [5,8,9].

The most frequently used method of obtaining CNFs is the carbonization of polymer precursors, mainly polyacrylonitrile (PAN) nanofibers obtained by electrospinning [10]. Electrospinning is one of the most frequently used methods of forming nanofibers, which gives more possibilities when selecting the polymer precursor itself and modifying the materials obtained [11]. A very important factor determining the potential of these nanomaterials in tissue engineering is that they perfectly mimic the extracellular matrix (ECM) architecture, and their degradation products are not cytotoxic. However, like carbon microfibers, their main disadvantage is their lack of bioactivity, and their hydrophobic nature due to the structure of carbon which causes limited cell adhesion [12]. Therefore, an important issue is the modification of the surface in a way that would allow improvements in both biocompatibility and affinity for cell structures and membrane receptors [13]. On the other hand, the modification may affect the deterioration or change of other properties, such as the electrical properties of CNFs, which, taking into account the possibility of combining these properties with electrical stimulation, may be unfavorable from the point of view of cell growth or proliferation. Carbon nanofibers can be modified by introducing functional groups on their surface as well as by introducing polymer, ceramic or other carbon-based layers with a proven effect on the growth of hard tissue cells. Oxidation in an oxidizing media, such as a mixture of sulfuric and nitric acids, or nitric acid itself, aims to introduce oxygen groups (such as carboxyl or hydroxyl groups) to the fiber surfaces, thus increasing the wettability of the surface and facilitating the process of cell adhesion and proliferation. As mentioned, CNFs can be modified with various polymers or biopolymers, including sodium hyaluronate. Sodium hyaluronate is characterized by excellent intrinsic biological recognition, it is biocompatible, non-immunogenic, and at the same time biodegradable. It is also a key molecule involved in many physiological and pathological processes [14,15]. Sodium hyaluronate interacts with cells through receptors on the cell surface, such as CD44 and CD168 [16]. Previous studies have shown that sodium hyaluronate accelerates the formation and deposition of bone matrix by stimulating the migration, adherence and proliferation of undifferentiated mesenchymal stem cells and inducing their differentiation into osteoblasts [15,17]. Research has shown that it can bind to several important proteins of the healing cascade, such as fibrin, fibrinogen, fibronectin and collagen [18]. It also has the ability to stimulate the expression of osteocalcin or osteopontin, as well as bone morphogenetic proteins and type I and type III collagen [17,18,19].

Modification of the surface with nanoparticles is also useful due to the possibility of nanostructuring of the surface and introducing certain changes to the surface topography which influences the stimulation of a cascade of consecutive intracellular events leading to the stimulation of cell adhesion. One such nanomaterial may be graphene oxide (GO), which, despite its lower thermal and electrical conductivity compared with graphene, has unique physical and chemical properties, such as a very large specific surface area (2600 m^2^ g^−1^), size, wettability, high adhesion and good biocompatibility, as well interacting better with other materials [20]. Recent studies have shown that GO-based materials have a wide range of applications in bone tissue engineering. GO has the ability to promote and enhance osteogenic differentiation, facilitate the growth and spread of bone cells, and increase the mechanical strength of bone tissue [15,21,22]. There are also reports indicating the stimulating effect of GO on cartilage matrix regeneration [12].

Silicon, as a relatively inert element, is involved in the formation of bone, cartilage and connective tissue, and participates in several important metabolic processes. Silicon-containing polymers are known as silicones, siloxanes or polysiloxanes, the structures of which are made up of repeating silicon-oxygen bonds [23]. Some of the organosilicon polymers, such as polysiloxanes, can be a precursor for the preparation of ceramics. Silicon-based ceramics are an interesting solution for the modification of nanofiber scaffolds for the regeneration of bone and cartilage tissue. A particularly interesting solution is the use of Si-based preceramic polymers (organosilicon polymers), which pyrolyze to silicon oxy-carbide (SiOC) and silicon carbide (SiC) through thermal treatment. The advantages of using polymer precursors to obtain ceramics as compared with traditional methods are the lower pyrolysis temperatures and the possibility of using polymer processing techniques such as dip coating and injection molding. Importantly, with regard to surface modification, the use of preceramics allows layers of different thickness to be obtained [24]. The advantages of a SiOC-based layer in the modification of scaffolds for bone tissue regeneration are, despite the high production temperature of these layers, the preservation of their amorphous nature and bioactivity. Moreover, SiOC materials are alkali-free and can therefore overcome some of the disadvantages, which have been discussed in the literature, of bioactive glasses with a high alkali content [23,25,26]. Ceramics based on SiOC in biological research are still poorly understood, and little work has been conducted on this topic. Thus, all research in this area is interesting and desirable.

Therefore, bearing in mind the potential of eCNFs as scaffolds for the regeneration of bone or cartilage tissue, in addition to the wide range possibility for modifications using chemical oxidation, applying layers in the form of polymers, ceramics or other carbon nanomaterials, and extending the characteristics of these materials is also justified from a biological point of view.

To date there has been no discussion in the literature of combining a nanofiber substrate based on carbon nanofibers with the above-mentioned modifications as a potential substrate for the regeneration of bone or cartilage tissue. Proving that it is possible to obtain such compositions, as well as indicating the importance of the proper characteristics of these materials in order to be able to conduct further, more advanced research in the future, is the overriding goal of this work. For this purpose, four types of CNF-based materials were obtained, which were oxidized in HNO_3_ and surface modified with GO, sodium hyaluronate and SiOC. The created composite substrates were subjected to physicochemical, structural and microstructural analysis using SEM, XPS, FTIR and goniometry. The influence of the modification on the porosity and electrical properties of the obtained composites in relation to the as-received CNFs was also assessed. The obtained samples were also subjected to preliminary biological in vitro tests when/while in contact with bone and cartilage cells.

## 2. Materials and Methods

The precursor nanofibers were obtained by the electrospinning method from a 10 wt.% of polyacrylonitrile (PAN, Zoltek Co., Bridgeton, MO, USA content 93–94% of acrylonitrile units, 5–6% of acrylate units and 1% of alkyl sulfonate) solution in *N,N*-dimethylformamide (DMF, from Avantor Performance Materials). A needle with a diameter of 1.1 mm was used with the nozzle positioned perpendicular to the collector, with 4 cm distance. The electrospinning process of nanofibrous mats was carried out by applying a voltage of 12 kV to the nozzle. The samples were formed for 30 min on the surface of aluminum foil. The conversion of PAN nanofibers into carbon nanofibers (CNFs) involved two steps, i.e., stabilization and carbonization. The stabilization process was carried out by heating the nanofibers at a rate of 3 °C/min to 250 °C, keeping them at this temperature for 30 min, then additional heating up to 270 °C and maintaining this temperature for another 20 min. The carbonization process was carried out in a quartz tube furnace by heating stabilized nanofibers in a nitrogen atmosphere to 1000 °C at a heating rate of 7 °C/min, without holding the samples at the final temperature.

The CNFs prepared in this way were divided into parts and then modified accordingly. Four types of surface modification of the obtained CNFs were prepared, i.e., oxidation in concentrated HNO_3_ (ox), modification with sodium hyaluronate (NaH) solution, graphene oxide (GO) and silicon oxycarbide (SiOC).

### 2.1. Oxidation in Nitric Acid

In order to carry out the oxidation process, the CNFs were put into a beaker and covered with 65% nitric acid (V) (HNO_3_) solution. The oxidation process was carried out in the fume cupboard for 1 h in the temperature range of 80–90 °C. In the next step, the nanofiber mats were washed several times with distilled water until checks with a pH-meter showed that their pH was neutral.

### 2.2. Modification Using Sodium Hyaluronate

To carry out the modification with sodium hyaluronate (NaH, Acros Organic, Lot: A0412454), a 0.5% solution of NaH in distilled water was prepared. The concentration of the NaH solution was selected experimentally with the primary consideration of good penetration of the carbon nanofiber mat with this solution. The prepared solution was placed on a magnetic stirrer for thorough mixing. The sodium hyaluronate solution was applied directly to the surface of CNFs after oxidation using a Pasteur pipette. An amount of 1 mL of the solution was applied to each side of the mat and the solvent was allowed to evaporate completely.

### 2.3. Modification Using Graphene Oxide (GO)

The graphene oxide modification (GO, >99 wt.%, length: 0.5–3.0 μm, thickness: 0.55–1.2 nm from Nanostructured & Amorphous Materials, Inc., Katy, TX, USA) to the surface of the carbon nanofibrous mat used a mixture of GO in a solution of ethanol/acetone/water (EAW), which consisted of 35 mg of GO sonicated in 12 mL the mentioned solution. The procedure for preparing the solution was described in previous articles [27,28]. Electrophoretic deposition (EPD) was performed on an experimental setup consisting of a DC power supply, electrolytic baths and two electrodes, one of which was the carbon nanofiber mat on which the graphene oxide coating was deposited.

### 2.4. Modification Using Silicon Oxycarbide (SiOC)

The purpose of the modification using polymethylphenylsiloxane resin (Lukosil 4102, produced in the Czech Republic Lučební závody a.s. Kolín) in the form of a solution in acetone was to obtain CNFs which would eventually be covered with silicon oxycarbide (SiOC). To this end, CNFs (after infiltration of Lukosil 4102) were subjected to a thermal treatment process to convert the resin into SiOC. Firstly, the prepared materials were allowed to evaporate the solvent in a fume cupboard, and were then placed in an oven for 1 h at a temperature of 150 °C. Finally, a thermal treatment process at 1000 °C was carried out with a heating rate of 5 °C/min and a nitrogen flow (20 L/h). 

As a result of the modification methods discussed in this chapter, five types of nanofiber materials were prepared:

- CNF—carbon nanofibers without modification;
- CNF_ox—carbon nanofibers after oxidation in HNO_3_;
- CNF_NaH—carbon nanofibers after oxidation in HNO_3_ and modification with sodium hyaluronate;
- CNF_GO—carbon nanofibers after modification with graphene oxide (GO);
- CNF_SiOC—carbon nanofibers after modification with silicon oxycarbide (SiOC).

### 2.5. Physicochemical Characterization of Modified Carbon Nanofibers

The morphology and microstructure of the CNFs, and CNFs after modification, was analysed using scanning electron microscopy (SEM) (NovaNanoSEM 200, FEI). In addition, X-ray energy dispersion spectroscopy (EDS) was applied to obtain an accurate determination of the elements present in the CNFs with and without modification. The diameters of nanofibers were measured based on SEM micrographs using Image J software. Data were analysed using statistical software (StatSoft Inc., Tulsa, OK, USA). The Mann–Whitney U test for multi comparison was used to assess statistical significance at *p* ≤ 0.05.

The surface chemistry of all CNFs mats was characterized by Fourier transform infrared (FTIR) spectroscopy. The FTIR spectra of the samples were recorded by means of a Tensor 27 spectrometer (Bruker Optics). The transmission FTIR spectra were registered in the range of 400–4000 cm^−1^ using the attenuated total reflection technique (ATR).

The contact angle of the obtained CNFs mats was measured by the sessile drop method using a DSA 25E analysis system and KRÜSS ADVANCE 1.6.1.0 software (KRÜSS, Hamburg, Germany). The contact angle was calculated by averaging the results of 20 measurements. The measurements were compared with an unmodified CNFs as a reference.

The scaffold mass was evaluated using scales (QUINTIX 125D 1CEU, Sartorius, Goettingen, Germany; precision of 0.00001 g). The membrane thickness was measured by a digital microscope (VHX-6000, Keyence, Japan). The average bulk density of carbon nanofibers was 1.8 g/cm^3^. Moreover, the surface porosity of all CNFs mats and fiber diameters in CNFs were established based on SEM microphotographs using Image J software. For porosity the thresholding of SEM images was performed. Thresholding is a type of image segmentation where the pixels of an image are changed to make the image easier to analyse. In thresholding, the images are converted from colour or grayscale into a binary image, i.e., one that is simply black and white. The porosity of the CNFs scaffolds was estimated using the gravimetric method (1) described in other papers [29,30].

(1) $$P=(1-\left(\frac{\rho }{\rho_{b}}\right))\cdot 100$$

where *P* is porosity [%], *ρ* is apparent density of nanofabric [$\frac{g}{m^{3}}]$, *ρ_b_* is average bulk density of the carbon nanofibers [$\frac{g}{m^{3}}]$.

The apparent density was calculated based on Equation (2):

(2) $$\rho =\frac{m}{d\cdot S}$$

where *m* is mass of the sample [g], *d* is sample thickness [cm] and *S* is sample area [${\mathrm{cm}}^{2}$].

The mass of the carbon nanofabric was evaluated using the internal scales of thermoanalyzer STA-449F3 (Netzsch, Selb, Germany; precision of 0.00001 g). The membrane thickness was measured with a digital microscope VHX-6000, Keyence, Japan. The average bulk density of CNFs was 1.81 g/cm^3^.

The electrical properties of CNFs scaffolds were examined using a four-point probe method using the T2001A3 4-point probe system (Ossila Ltd., Sheffield, UK). The main purpose of studying the electrical properties of CNFs was to assess the influence of the type of modification on the conductive properties of composite samples. The samples had the form of rectangles (10 × 5 mm) cut directly from the mats. At least three separate specimens were measured for each type of scaffold, and 25 measurements were taken for each sample. During the measurements a constant pressure of 60 g was applied to the contact probes (r = 0.24 mm, distance = 1.27 mm). The surface resistivity and volume conductivity were calculated using Equations (3) and (4):

(3) $$R_{S}=\frac{C\;\pi }{ln\left(2\right)}\;\frac{\Delta V}{I}$$

(4) $$\sigma =\frac{l}{R\;A}$$

where *R_s_* is surface resistivity, *C* is geometric correction factor, Δ*V* is voltage loss between the probes, *I* is the applied current, *σ* is volume conductivity, *l* is the distance between the probes, *R* is measured resistance, *A* is the cross-sectional area of the sample [31].

### 2.6. Biological Influence of CNF-Preliminary Studies 

For the assessment of the cytotoxicity of materials the live/dead cell staining method was applied. This fluorescesnt kit uses two dyes i.e., calcein AM (C1359, Sigma-Aldrich, Saint Louis, Germany) and propidium iodide (P8464, Sigma-Aldrich, Germany). The non-fluorescent calcein AM, after penetration through the cell membrane, is converted by esterase in the cytoplasm of living cells into a green fluorescent, membrane-impermeable compound. The second dye, propidium iodide, after penetrating into the dead cells binds with nucleic acids and stains them red.

To carry out biological studies, two types of cell line were used, i.e., osteosarcoma cell lines MG-63 (European Collection of Cell Cultures, Salisbury, UK), and human chondrocyte cell line CHON-001 (ATCC CRL-2846). After sterilization with ultraviolet radiation (UV-C) for 30 min, the tested nanofibers were placed in tissue culture test plate 48 wells (Nunclon), in which MG-63 cells were seeded at a density of 20,000 per well in a culture medium (MEM) containing 10% fetal calf serum, 5% penicillin/streptomycin, 1% amino acid and 1% sodium pyruvate (all from PANBiotech, Vienna, Austria).

CHON-001 cells were seeded at a density of 10,000 per well in chondrocyte growth medium CGMTM (Lonza, catalog #: CC-3216). Both types of cells were grown on CNFs mats, both modified and unmodified, for 72 h and stained with fluorescent dyes: calcein and propidium iodide. 

A fluorescence microscope (Zeiss Axiovert 40, Carl Zeiss, Jena, Germany) was used to observe the cells in contact (both MG-63 and CHON-001 cells line) with the materials. Based on captured microscope images, a qualitative assessment of changes in cell morphology on the tested materials was carried out. At the same time, Image J software was used for the quantification of live and dead cells and calculation of the surface area occupied by cells on the CNFs’ surface before and after surface modification. For this purpose, thresholding of cell images on the samples was performed.

## 3. Results and Discussion

### 3.1. Morphology and Microstructure of CNFs

**Figure 1:**
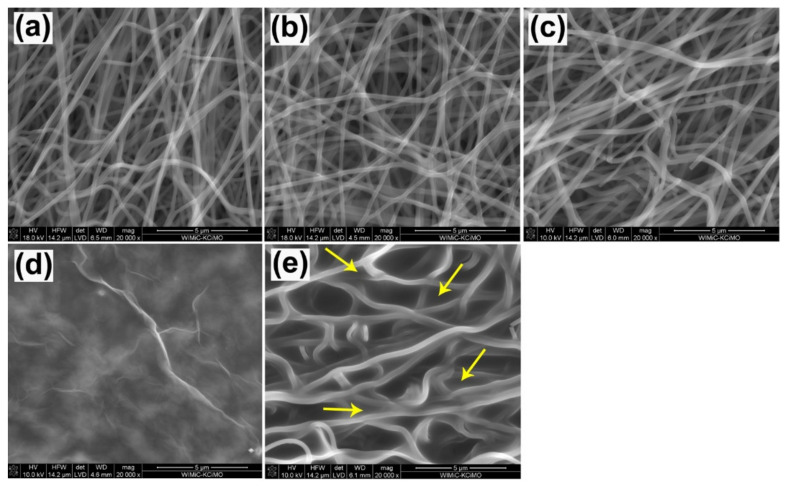
SEM microphotograph of CNFs before (**a**) and after surface modification in HNO_3_ (**b**), in sodium hyaluronate (**c**), using GO (**d**) and using silica oxycarbide (**e**).

The morphology and microstructure of CNFs before and after surface modification were presented on microphotographs (Figure 1).

**Figure 2:**
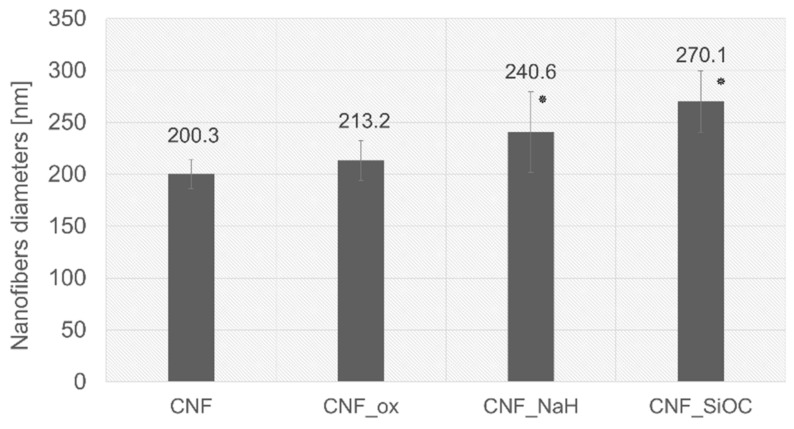
Comparison of mean values of nanofiber diameters, *—result statistically different from CNF (*p* < 0.05) based on the Mann–Whitney U test.

Morphology analysis based on SEM images showed no major differences between the CNFs sample and the materials after oxidation (CNF_ox). Similarly, no major differences were observed between the materials after oxidation (CNF_ox) and after modification with sodium hyaluronate (CNF_NaH) (Figure 1a–c). These materials retained a fibrous microstructure with randomly distributed fibers. Analysis of the diameters of these three types of nanofiber showed slight differences between them (Figure 2). In particular, there were no significant differences between unmodified CNFs and after oxidation (CNF_ox). This was also confirmed by the statistical analysis which showed no statistical differences between these samples. The lack of significant differences, in terms of both microstructure and diameter, between CNFs and CNF_ox indicated no negative influence of the oxidant (HNO_3_) on the structure of CNFs. Among various oxidants, nitric acid was favourable because its processing could be easily controlled by adjusting acid concentration, treatment temperature, and time [32,33]. Thus, it seems that the proposed conditions of fiber oxidation were not aggressive enough to cause degradation of the surface layers of CNFs. The literature indicates several conditions of the oxidation process with nitric acid which may contribute to the destruction of the surface of carbon materials. Most often, when using concentrated HNO_3_, the time at which changes in the microstructure and structure of the material are first observed is over 5 h [33,34]. In the case of nanofibers modified with sodium hyaluronate (CNF_NaH), a slight increase in the diameter of nanofibers was observed in relation to the unmodified nanofibers (Figure 2). This increase was most likely due to the presence of a thin layer of sodium hyaluronate around the individual nanofibers.

In the case of modification with graphene oxide (CNF_GO), the morphology of the obtained substrates differed significantly from that of the CNF surface (Figure 1d). The SEM images do not show the characteristic fibrous forms of nanofibers, but a homogeneous layer covering the surfaces of the CNF. The presence of some streaks and flakes on the surface of CNF_GO suggested the presence of graphene layers on the surface of the samples (Figure 1d arrows). Visible wrinkles were attributed to the overlapping of single graphene layers, as well as the presence of oxygen groups and structural defects present on the GO surface [35,36]. These phenomena affected the change in the hybridization of carbon atoms from sp^2^ to sp^3^, thus causing the carbon atoms to deviate from the original plane, which was visible at the atomic level in the form of folds [36]. Similar structures were observed in earlier research [28]. It was not possible to determine the diameter of the nanofibers for this sample. Modification with SiOC obtained as a result of thermal decomposition of polysiloxane, influenced the morphology of nanofibers. The nanofiber microstructure can still be observed in the photomicrographs, with the difference that the smaller pores formed as a result of the crossing of individual nanofibers were probably filled with a layer of SiOC (Figure 1e). The shape of the pores changed, and there was also a significant increase in diameter of about 35% compared with the unmodified CNFs.

### 3.2. Porosity of CNF

**Figure 3:**
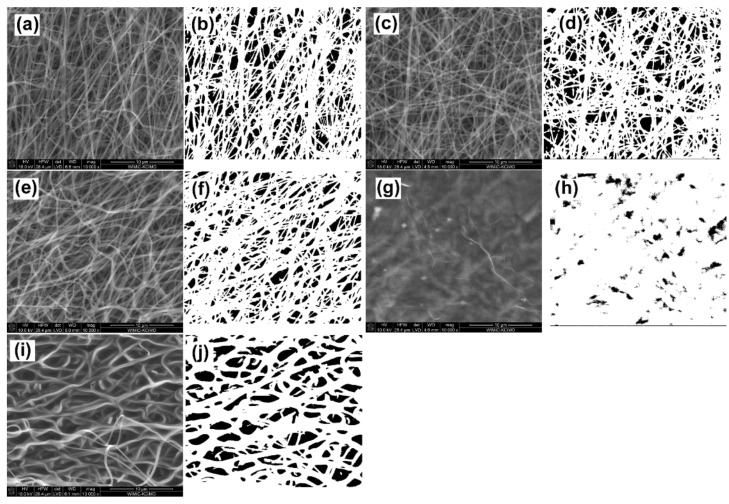
SEM images before and after thresholding for (**a**,**b**) CNFs; (**c**,**d**) CNF_ox, (**e**,**f**) CNF_NaH, (**g**,**h**) CNF_GO, and (**i**,**j**) CNF_SiOC.

Porosity is an important parameter when considering the application of CNFs for the regeneration of hard tissues such as bone or cartilage. The surface porosity of CNFs with and without modification was examined/determined using SEM microphotographs and ImageJ software. The SEM images and their thresholding are presented in Figure 3.

**Figure 4:**
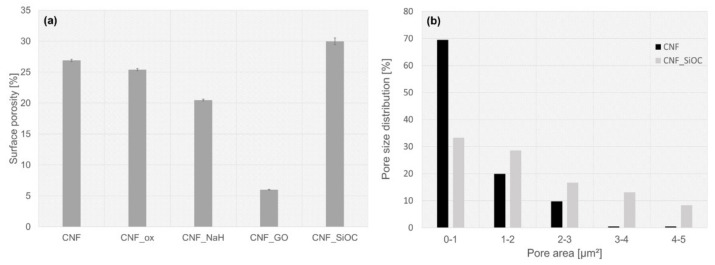
Surface porosity calculated based on SEM thresholding (**a**); Pore size distribution on the surface of CNF and CNF_SiOC samples (**b**).

The surface porosity varied depending on the type of modification used. The CNFs and CNT_ox samples were the most similar, while for the sample after modification with sodium hyaluronate, the porosity was slightly lower, although the pore shapes were comparable to those for the CNFs and CNF_ox samples (Figure 3a–f and Figure 4). The slightly lower porosity in the case of CNF_NaH was most likely due to the increase in diameters of nanofibers caused by coating them with polymer. The slightly lower porosity in the case of CNF_NaH was most likely due to the increase in diameter of nanofibers caused by them being coated with polymer, which is documented in Figure 2. The most significant difference in porosity was observed for the CNF_GO sample (Figure 4). Graphene oxide created a continuous layer on the CNFs’ surface, making the fibrous form of the material invisible. This contributed directly to the surface porosity. The dark spots in the images were the result of roughness or certain depressions in the surface rather than of pores (Figure 3h). The sample modified with SiOC had the highest porosity. In the case of this sample the fibrous form was retained, which proved that the preceramic, prior to the thermal treatment towards SiOC formation, infiltrated through the entire thickness of the CNFs without isolating the nanofiber surface, which was the case with the CNF_GO sample. During the thermal transformation of polysiloxane silicon oxycarbide, coatings were formed on the surface of nanofibers, which was confirmed by an increase in the diameter of the fibers (Figure 2). In addition, layers of SiOC were also formed that filled the spaces between intersecting nanofibers (Figure 1e, arrows), causing the shape of the pores to change; they became more curved but the sample surface retained its fibrous form (Figure 3j). Interestingly, the presence of silicon oxycarbide in the form of layers on the surface of the CNF did not significantly affect the porosity of the surface in comparison with the reference sample (CNFs) (Figure 4a). While the mean surface porosity for CNF_SiOC sample was comparable to the control sample, the pore size distribution was different, shifting towards larger pores (Figure 4b).

The porosity of the CNF-based samples calculated using the gravimetric method are presented in Table 1. The total porosity was similar for all samples. These results indicate that the modification of the CNFs sample with graphene oxide occurred only on the surface of the sample. For this sample the total porosity was at a similar level to that in the other samples, while analysis of its surface porosity showed the greatest variation from the other samples. For the CNF_SiOC sample, although silicon oxycarbide influenced the shape and distribution of the pore size, it did not substantially change the total porosity of the sample, and it still retained the form of a 3D scaffold.

### 3.3. Surface Chemistry of CNF before and after Modification

- FTIR

**Figure 5:**
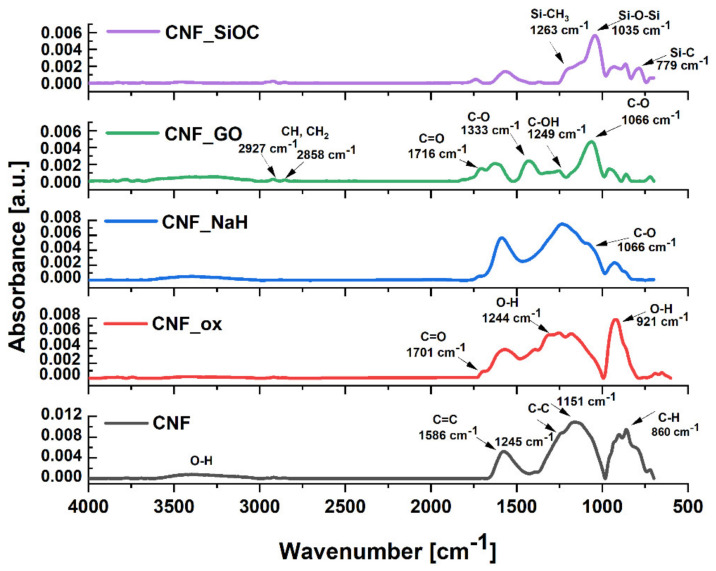
FT-IR spectra of the CNF before and after modification.

The surface chemistry of modified and unmodified CNFs scaffolds was assessed by FTIR. The set of FTIR spectra for the as-received CNF and modified CNF are shown in Figure 5. The bands of CNFs spectra at 1245 cm^−1^ and 1151 cm^−1^ were related with the C–C stretching vibrations, and the band at 860 cm^−1^ was associated with the vibration of the C–H groups in the aromatic ring. The peak at around 1586 cm^−1^ was attributed to the vibrations of the C=C group of graphene rings which was caused by C=C stretching and bending vibrations of the graphene back bone [37,38]. In turn, a wide band in the range of 3500–3066 cm^−1^ was related to oscillation vibrations of the O–H groups. A weak band at 2916 cm^−1^ and another at 2846 cm^−1^ were associated with C–H bond stretching vibrations arising from hydrogen atoms [39]. 

After treating the CNFs in HNO_3_, the IR spectrum was partially similar to the spectrum of unmodified CNFs. There were a number of slightly shifted bands in the spectrum, i.e., 1571, 1244, 1176 and 864 cm^−1^, which were derived from the vibrations of the C=C, O–H, C–C and C–H groups, respectively [40]. Compared with untreated CNFs, the acid-treated CNFs exhibited a new peak at about 1701 cm^−1^, which was assigned to the C=O stretching vibrations of carboxylic/lactone groups [41]. The characteristic intense band for CNFs after treatment in HNO_3_ at a wavenumber of 921 cm^−1^ was associated with the vibrations of the O–H group. The intensity of the wide band in the range of 3500–3066 cm^−1^ from O–H bonds did not show any noticeable changes.

Some subtle changes were observed in the spectrum of the CNF_NaH, indicating the presence of sodium hyaluronate. Compared with sample CNF_ox, these changes were visible in the wavenumber range 1020 to 1360 cm^−1^, the presence of the band at the wavenumber of 1066 cm^−1^ coming from the skeletal vibrations of C–O and C–C provided particularly strong evidence of the presence of sodium hyaluronate [42]. The intensity of the bands at 915 cm^−1^ related to the O–H groups also changed. There was an increase caused by modification with sodium hyaluronate. The bands characteristic for sodium hyaluronate located at the wave numbers 1616 cm^−1^ and 1556 cm^−1^, related to Amide II and (δ (-NH_2_)) respectively, were probably invisible due to the characteristic band in this area for carbon nanofibers being derived from the C=C group [42].

The spectrum of the CNF_GO nanofibers confirmed the presence of oxygen-containing functional groups characteristic of GO. This spectrum presented characteristic bands at 1066 cm^−1^ assigned to the C–O stretching vibrations of C–O–C, and at 1249 cm^−1^ corresponding to C–OH stretching of alcohol groups [2]. The band at 1333 cm^−1^ was assigned to C–O stretching vibrations. The peak at around 1641 cm^−1^ was attributed to C=C stretching from the unoxidized graphitic domain, while the band at 1716 cm^−1^ was assigned to C=O stretching vibrations of carboxyl groups [43,44]. The IR peak corresponding to 2927 cm^−1^ was due to the asymmetric CH_2_ stretching of GO. The broad band at 3000–3500 cm^−1^ was due to the carboxyl OH stretching mode from COOH and H_2_O [45,46].

FTIR spectra for the sample modified with silicon oxycarbide showed characteristic bands indicating the presence of silicon at the surface of CNF_SiOC. The spectrum of this sample displayed distinct stretching modes characteristic of Si-C and Si–O–Si bonds at approximately 779 and 1035 cm^−1^, respectively [47,48]. In addition, the band at higher wavenumbers (about 1263 cm^−1^) was attributed to the Si–CH_3_ stretching mode [47].

**Figure 6:**
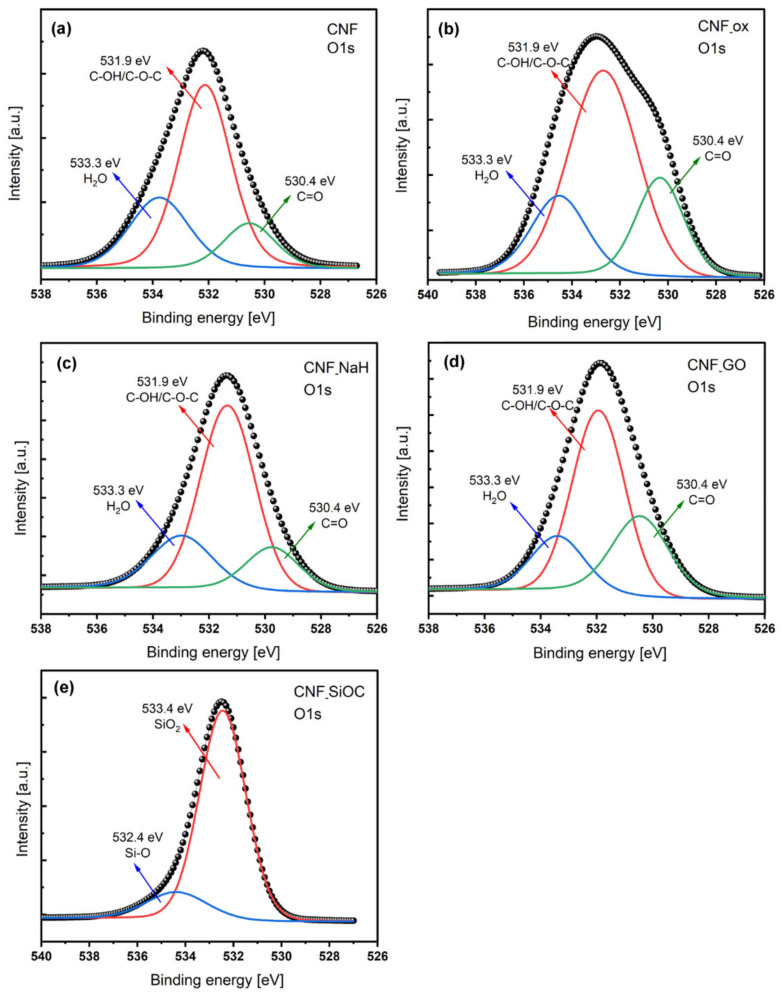
XPS spectra of carbon nanofibers before and after surface modification. Deconvoluted O1s (**a**–**e**).

The surface elemental composition of the modified CNFs was also characterized by XPS as shown in Figure 6a–e. This graph shows the signals at binding energy 532 eV characteristic for O1s. Table 2 shows the chemical composition of the CNFs before and after surface modification.

Elemental analysis showed that the unmodified sample (CNFs) contained the highest amount of carbon (85.8 at.%) and nitrogen (7.3 at.%) of all the tested samples. These nanofibers also contained 6.9 at.% of oxygen. Their presence in the CNF resulted from the chemical composition of the PAN nanofiber precursor, and also from the stabilization process, which was carried out in air atmosphere. The modification process significantly changed the amount of individual elements and introduced additional elements depending on the composition of the modifier. After the oxidation process of CNFs in HNO_3_ (CNF_ox), the carbon content decreased to 79.0 at.%, whereas the oxygen content increased to 18.1 at.% and the nitrogen content decreased from 7.3 at.% to 2.9 at.% (Table 2). Modification of the CNFs in sodium hyaluronate (CNF_NaH) caused a decrease in the amount of carbon to the value of 68.4 at.% and an increase in the amount of oxygen to the value of 26.4 at.%, in comparison with the unmodified CNFs and also CNFs after oxidation (CNF_ox). Taking into account that for the modification of CNF with sodium hyaluronate nanofibers after oxidation in HNO_3_, the increase in the amount of oxygen and nitrogen resulted from the presence of hyaluronate on the surface of the CNF_NaH. The presence of Na on the surface also confirmed the presence of sodium hyaluronate on the surface of these fibers. Graphene oxide obtained by the Hummers method and its modifications contains a large quantity of oxygen groups, which increased the amount of this element in sample CNF_GO (25.0 at.%) [44]. The presence of silicon oxycarbide on the surface of sample CNF_SiOC was evidenced by the presence of Si in the XPS analysis.

The XPS method allowed not only the determination of the atomic content of individual elements in the sample, but the identification of functional groups on the CNFs’ surface before and after modification. The main band for as-received CNFs at 284.6 eV was derived from sp^2^ carbon atoms occurring in the form of C=C or C–C bonds [49]. Depending on the type of sample (e.g., CNF_SiOC), this binding energy may also have involved the C–Si–O bond (284.88 eV) [50]. This band was also deconvoluted into bands where carbon atoms were present in oxygen-containing groups around at 285.8 eV, 286.8 eV, 288.2 eV, and 290.3 eV, corresponding to C–O–C and/or C–O, C=O, O=C–OH and O=C–O–O–, respectively. In the as-received CNFs, three oxygen (O1s) bonds (Figure 6) were distinguished, at 530.8 eV corresponding to group C=O, at 532.4 eV corresponding to functionalized oxygen-containing groups, such as C–OH or C–O–C and at 534.0 eV assigning moisture [49,51]. The presence of these groups was visible in every sample with the exception of sample CNF_SiOC. The spectrum of O1s for CNF_SiOC given in Figure 6e showed two fitting peaks at 532.44 and 533.37 eV related to Si-O and SiO_2_ bonds [50,52,53]. Deconvolution of the O1s band for CNF, CNF_ox, CNF_NaH and CNF_GO showed the highest increase in C–OH and C–O–C groups for sample CNF_NaH compared with CNF (Figure 6a–d). Concentration of C=O groups was highest for CNF_GO (28.2 at.%) and for CNF_ox (21.7 at.%) in comparison with as-received CNFs (14.4 at.%). 

An energy peak at 102.97 eV for CNF_SiOC was fitted to Si 2p, suggesting that the presence of Si–O or O–Si–O bonds were detected at the surface of sample [52,54]. The presence of these functional groups was also confirmed by the bands visible in the FTIR spectra of this sample (Figure 5).

### 3.4. Wettability of Modified CNF

**Figure 7:**
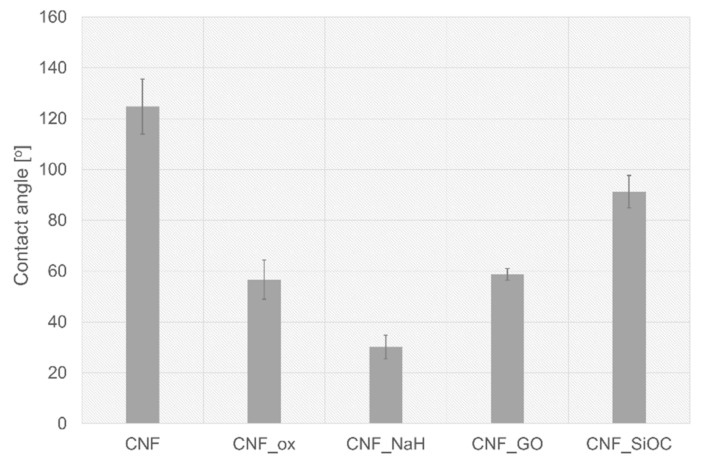
Water contact angle values for as-received CNF, and CNF after modification.

The presence of chemical groups on a CNFs’ surface had a significant impact on the wettability (Figure 7).

The lowest wettability was observed for as-received CNFs (θ = 124.8 ± 10.8°). The hydrophobic nature of CNFs is due to their structure, where carbon is almost purely based on aromatic, non-polar sheets, so that interaction with extremely polar molecules such as water is very weak. The highest wettability was observed for CNF_NaH (θ = 30.2°) for which the highest number of oxygen-containing groups (26.4%) of all analysed samples was observed (Table 2). Sodium hyaluronate is the salt of hyaluronic acid (HA) which is a linear chain containing repeating disaccharide units consisting of N-acetyl-D-glucosamine and D-glucuronic acid connected by ß 1,3-glycosidic bonds [14]. Disaccharide units have a large number of functional groups that are hydrophilic in nature such as hydroxyl, carboxyl and acetamido [14,55]. Moreover, in an aqueous solution, NaH forms salts with Na+ and others which are highly hydrophilic and consequently surrounded by water molecules [14]. XPS analysis for this sample showed the highest number of C–OH/C–O–C (62.0 at.%) groups among all the tested samples. An increase in wettability was also observed for CNFs samples after modification with graphene oxide (CNF_GO) and oxidization in HNO_3_ (CNF_ox). The wettability for these samples was 58.7 ± 2.3° and 56.7 ± 7.7°, respectively. The decrease in the contact angle for these samples in comparison with CNF was directly related to the presence of hydrophilic oxygen groups, which was proven in both FTIR and XPS tests (Figure 5 and Figure 6). For these two samples, carbonyl groups were the dominant type of functional groups. The presence of silicon oxycarbide on the CNFs’ surface caused a significant increase in wettability in relation to the as-received CNFs (from θ = 124.8 ± 10.8° do θ = 91.3 ± 6.4°). However, compared with the samples modified with HNO_3_, GO and sodium hyaluronan, the contact angle for this sample was significantly higher and was defined as a hydrophobic material. This is due to the hydrophobic nature of SiOC ceramics [56,57].

### 3.5. Electrical Properties of Modified CNF

**Figure 8:**
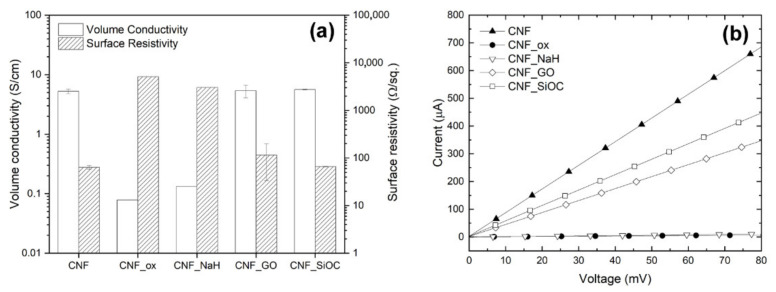
(**a**) Volume conductivity and surface resistivity of various types of modified carbon nanofibers. (**b**) Corresponding current-voltage curves.

The volume conductivity and surface resistivity of CNFs measured using the four-point probe method are shown in Figure 8a, and the corresponding I-V curves are shown in Figure 8b. As expected, as-received CNF and samples not subjected to the oxidative treatment (CNF_GO and CNF_SiOC) were characterized by similar values of conductivity around ~5.5 S cm^−1^. These modifications had no significant effect on the volume conductivity of scaffolds because they did not involve structural changes to CNF nor did they disrupt the integrity of the conduction web. In contrast, the oxidation of CNFs in HNO_3_ caused a significant decrease in conductivity of up to 0.078 S cm^−1^, resulting from the interruption of the sp^2^ hybridized network of carbon atoms and thus restricting the availability of charge carriers in the material. In turn, the modification of oxidized CNFs with sodium hyaluronate (CNF_NaH) caused an enhancement of conductivity to 0.132 S cm^−1^, probably due to the additional contribution towards ion conduction from sodium hyaluronate [58].

In general, the surface resistivity of the samples was directly connected to their volume conductivity The sole exception was the CNF_GO specimens, which, because of the presence of an insulating layer of GO on their surface, had higher surface resistance (R_s_ = 115.4 Ω/sq) as compared with the neat CNFs (R_s_ = 63.7 Ω/sq), but significantly lower surface resistance than the CNF-ox and CNF_NaH samples. The samples coated with SiOC had a value of R_s_ = 65.7 Ω/sq, which was very close to the unmodified CNFs, due to the fact that the ceramic layer did not cover the entire surface of the CNFs and was highly porous allowing for relatively good contact between the probes and the surface of the nanofibers. The samples subjected to the oxidative treatment were characterized by the largest surface resistivities of 5108.0 and 3021.5 Ω/sq for CNF_ox and CNF_NaH, respectively.

### 3.6. Biological Properties of Modified CNF in Contact with Osteoblast and Chondrocytes Cell Lines

**Figure 9:**
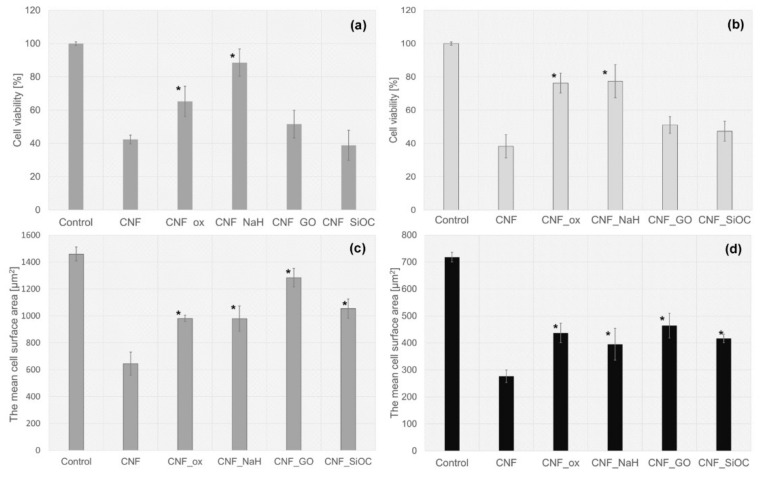
Viability of (**a**) osteoblast-like (MG-63) cells and (**c**) human chondrocyte (CHON-001) cells in contact with CNF before and after surface modification. The average MG-63 (**b**) and CHON-001 cells (**d**) surface area in contact with materials. The Mann-Whitney U test for multi comparison was used to assess statistical significance at * *p* ≤ 0.05 vs. as-received CNF.

**Figure 10:**
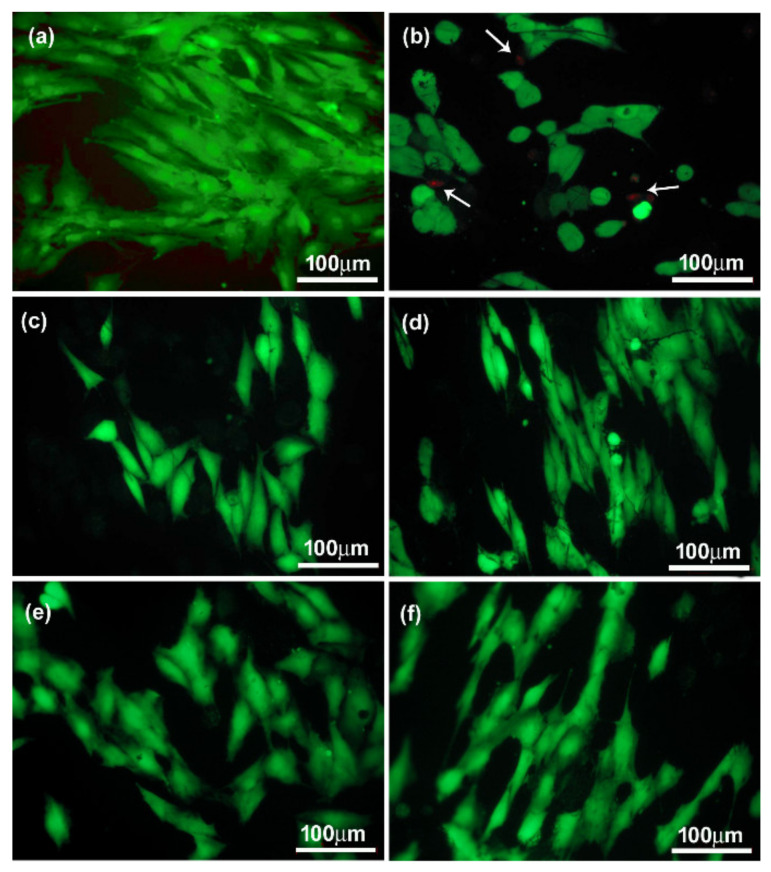
Merged microphotographs of osteoblast-like (MG-63) cells stained with calcein AM/propidium iodide grown on control (PS) (**a**) and CNF before (**b**) and after surface modification: (**c**) CNF_ox, (**d**) CNF_NaH, (**e**) CNF_GO and (**f**) CNF_SiOC.

**Figure 11:**
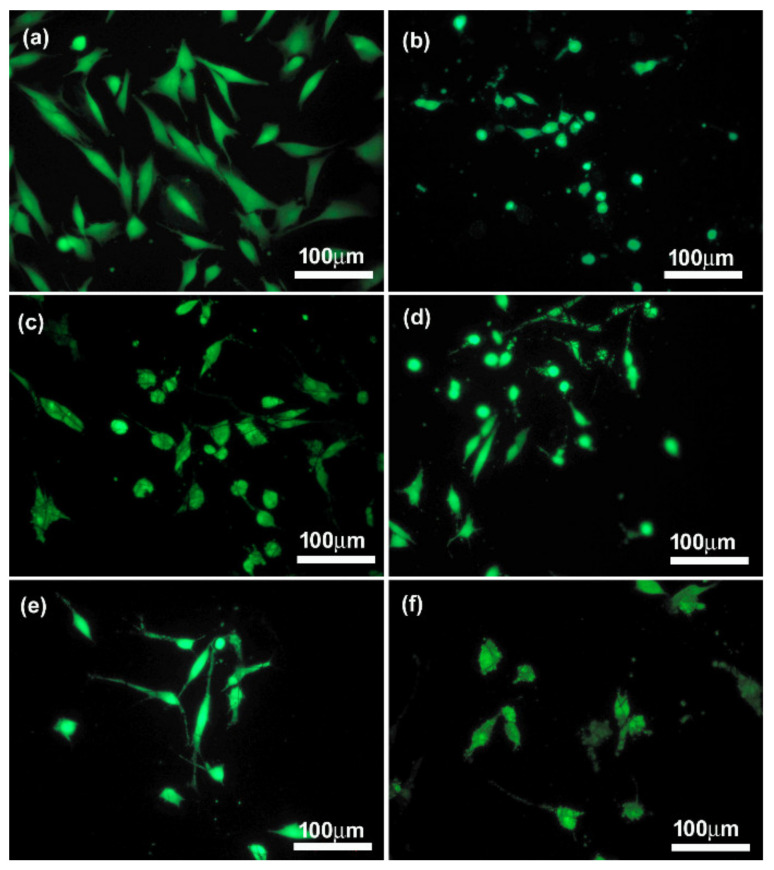
Merged microphotographs of human chondrocyte cell line (CHON-001) stained with calcein AM/propidium iodide grown on control (PS) (**a**) and CNF before (**b**) and after surface modification: (**c**) CNF_ox, (**d**) CNF_NaH, (**e**) CNF_GO and (**f**) CNF_SiOC.

In vitro studies were carried out on two types of cells, namely the osteoblast-like (MG-63) cells and on chondrocyte cell lines CHON-001. The assessment of biocompatibility was performed, both quantitatively and qualitatively, based on the images from the fluorescence microscopy of cells on the tested materials stained with calcein and propidium iodide (Figure 9a–d). The evaluation was made after 72 h of cultivation. In order to determine the number and surface area of cells from microscopic images, the ImageJ program was used. The number of cells on the surface of the CNFs before and after modification was presented as the percentage of cells in relation to the control sample, i.e., polystyrene (PS) well. Preliminary in vitro studies of both MG-63 and CHON-001 cell lines (Figure 10 and Figure 11) showed the fewest living cells were present on the surface of unmodified nanofibers (CNFs). Additionally, in fluorescence microscope images, the presence of dead cells was observed, especially in the case of MG-63 cells (Figure 10b, arrows). The introduction of modifications to the surface of the CNFs caused, in almost all cases, an increase in the number of cells of both chondrocytes and osteoblasts as compared with the unmodified sample. In the case of osteoblasts, the number of cells on the surface of the CNF_SiOC samples was similar to that of the CNFs, while for sample CNF_GO, the number of cells was slightly higher than for CNFs, although it was not statistically significant (Figure 9a). A similar situation was observed for chondrocytes, although the number of cells grown on samples CNF_SiOC and CNF_GO was slightly higher than for CNFs, these were not statistically significant differences compared with the unmodified sample (Figure 9c). The greatest increase in the number of cells (both MG-63 and CHON-001 cell lines) was observed for samples CNF_ox and CNF_NaH (Figure 9a,c).

Interestingly, when analysing the average cell surface area on the surface of the tested samples, it was observed that each modification had a positive impact on the increase in this parameter in relation to the unmodified sample (CNFs) (Figure 9b,d). The mean cell surface area of the control sample (PS) was 1460 ± 450 µm^2^ for MG-63 cells and 718 ± 450 µm^2^ for CHON-001 cells. The average size of cells on unmodified CNF was 644 ± 287 µm^2^ and 275 ± 83 µm^2^ for MG-63 and CHON-001 cell lines, respectively; this was smaller than the cell size on the modified nanofibers. By improving the hydrophilicity of the material, the degree of cell adhesion was increased. The mean cell surfaces on the modified nanofibers differed statistically from the cells on the surface of the CNFs sample. For all modifications, the degree of cell spreading related directly to their surface area was higher than for unmodified CNFs. This was probably the result of the increase in wettability of most samples, especially CNF_ox, CNF_NaH and CNF_GO, which resulted in a higher affinity of the surface of these materials to cells. Good surface wettability of biomaterials is one of the main factors that positively influences the cellular response [59]. As can be seen from the presented results, high surface wettability had a particularly significant effect on the rate of the cell proliferation, which was observed for sample CNF_NaH for which this parameter was the highest, and the number of both osteoblast and chondrocyte cells after 72 h of culture was also the highest (Figure 9a,c). Surface wettability was one of the factors influencing cell growth, but it was, of course, not the only one. The increase in cell count for sample CNF_NaH was most likely due to the nature of sodium hyaluronate, which is known to be a biocompatible linear glycosaminoglycan, being a key molecule involved in a variety of physiological and pathological processes. It can interact with receptors on the surface of the cell membrane, stimulating the migration, proliferation or even differentiation of cells into, e.g., bone cells [14,17,60]. Earlier studies have also shown the positive effect of sodium hyaluronate on chondrocytes [61,62,63]. These studies have shown that exogenous hyaluronic acid and its derivatives, such as sodium hyaluronate, are incorporated into the articular cartilage where it can have a direct biological effect on chondrocytes via CD44 receptors [62,63]. Interestingly, in the case of CNF_ox and CNF_GO, the wettability was similar, i.e., at the level of about 60° (Figure 7), but the effect on the number of cells was different (Figure 9a,c). In the case of sample CNF_GO, more oxygen (Table 2) than for CNF_ox was observed, although, for this sample (CNF_ox), carbonyl (C = O) was the dominant oxygen group (Figure 6). The main difference between the two samples was related to their surface morphology (Figure 3c,d,g,h). The surface of the GO modified sample (CNF_GO) was smoother and less porous than the CNF_ox sample, which did not differ significantly in terms of microstructure from the CNF or CNF_NaH samples (Figure 1 and Figure 3). It seems that, while higher surface porosity affected the number of cells adhering to the surface, this parameter had a smaller effect on cell spreading (Figure 9a,b). The mean cell surface area of the CNF_GO sample, especially for bone cells, was higher than that of the CNF_ox or even CNF_NaH sample (Figure 9b). It is probable that a high surface development of graphene oxide, and thus a large number of active sites capable of binding with cell membrane receptors, made the cells adhere more strongly to the substrate. The literature shows the ability of GO to facilitate the growth and spread of bone cells [64]. In addition, electrical properties are a factor that may have an influence on the increase in the surface area of cells, especially bone tissue cells. Graphene oxide, despite the fact that its conductivity is lower than that of graphene itself, shows properties on the border of semiconductor and insulator, depending on the degree of reduction of the oxygen groups [65,66]. In the case of the tested samples, the volume conductivity of CNF_GO was about 5.4 S/cm, which was much higher than that of samples CNF_ox and CNF_NaH (Figure 8a). However, it was comparable with the volume conductivity for the unmodified CNFs sample (~5.3 S/cm). In addition, the surface resistivity, although higher than for unmodified CNF, remained significantly lower than for samples CNF_ox and CNF_NaH. Thus, it could be argued that it is not a single parameter that determines the cellular response, but the appropriate synergy between the different parameters. In the case of sample CNF_GO, good surface wettability, high surface development and smoothness may have had a decisive influence on the cellular response.

The presence of silicon oxycarbide did not significantly affect the number of cells adhering to the nanofiber surfaces in either the MG-63 or CHON-001 cell lines. However, the presence of Si-ceramics on the CNFs’ surface influenced the degree of cell spreading in comparison with the unmodified sample, despite the cell morphology not differing from the cell morphology on the surface of the control sample (PS) (Figure 10). The cells on the surface of sample CNF_SiOC were well flattened, elongated, not shrunken or spherical as they were in the case of cells on the surface of the unmodified sample. Additionally, the presence of dead cells was not observed in the microscopic images. The presence of SiOC on the CNFs’ surface slightly lowered the contact angle compared with the as-prepared sample, but it was still at the level characteristic of materials defined as hydrophobic (Figure 7). For this sample, however, surface porosity increased, and the pore distribution and shape changed, compared with the original sample. The electrical conductivity for this sample, in turn, was at the level of the starting sample. The presence of Si in the structure and on the surface of the sample was likely to be one of the main factors influencing the spread and shape of cells (Table 2). The presence of oxygen-silicon groups and carbon-silicon was confirmed in both XPS and FTIR analysis (Figure 5 and Figure 6). The role of silicon in the skeletal and cartilage systems is known as a relatively inert element involved in the formation of bone, cartilage and connective tissue and is involved in many important metabolic processes [23].

## 4. Conclusions

The results demonstrate the potential of electrospun carbon nanofibers as a 3D substrate in tissue engineering. Their fibrous nature, porous microstructure and interesting physicochemical properties make them a basis for obtaining scaffolds for tissue regeneration. This study shows that CNFs can be successfully modified with various materials such as GO, SiOC and sodium hyaluronate with proven properties for stimulation/regeneration of bone and cartilage tissue. A very big advantage of using CNFs as scaffolds is its resistance to many chemicals and high temperatures, therefore these materials can be modified both in acids, such as HNO_3_, which improve the wettability of these materials, and in the case of polysiloxane, pyrolyzed at 1000 °C in a protective atmosphere to obtain ceramic layers (SiOC). This significantly increases the range of applicability of these materials, as well as allowing the production of a wide variety of products. In this work, four modifications of the surface of carbon nanofibers were applied: oxidation in HNO_3_, overlaying sodium hyaluronate layers, GO layers and SiOC layers. Numerous tests were performed to establish the physicochemical properties of the four types of modified CNFs. When analysing all modified CNFs, an increase in wettability was observed for all samples, and in particular for CNF_ox, CNF_NaH and CNF_GO as compared with the unmodified CNFs. The presence of only one modifier, namely graphene oxide, changed the morphology of CNF-modified surfaces. For the remaining samples, the modifications did not change the fibrous nature of the surfaces of the tested samples. The modifications did not affect the total porosity of the CNFs, whereas, in the case of GO and SiOC, they changed either the surface porosity or the size, distribution and shape of the pores. Modifications affected the surface chemistry, in the case of CNF-ox, CNF_NaH and CNF_GO contributing to an increase in the number of oxygen groups. In the case of the CNF_NaH sample, the C–OH and C–O–C groups dominated, while in the CNF_GO and CNF-ox samples, the C=O groups were dominant. In the CNF_SiOC sample, the presence of Si in the form of Si–O or O–Si–O groups was observed. The study of the electrical properties showed that the dominant influence on volume conductivity was functionalization of CNFs in nitric acid, which caused the conductivity to drop from 5.3 S/cm to 0.08 S/cm for the CNF_ox sample, and to 0.13 S/cm for the CNF_NaH sample. For samples modified with GO and SiOC, the values of volume conductivity and surface resistivity were at the level of the as-received CNFs.

Initial biological tests with the culture of MG-63 and CHON-001 cell lines showed no cytotoxicity for all samples tested. Overall, it was concluded that the modification of each sample improved the cellular response. Differences were observed in the number of cells and the area they occupied on the surface of modified CNFs. The results showed that two samples in particular, namely CNF-ox and CNF_NaH, which had the highest wettability and the lowest electrical conductivity, increased the number of both MG-63 and CHON-001 cells after 72 h in cell culture. However, the adhesion of cells and their spread on surfaces was inferior or comparable to other modified samples. Much better cell spreading was observed on the surface of the CNF_GO sample, which differed from CNF_ox and CNF_NaH mainly due to the surface morphology (smoother) and higher electrical conductivity while maintaining comparable wettability to the above-mentioned samples. Similarly, for the CNF_SiOC samples, a better spreading of cells, especially of osteoblast-like cells, was observed on the surface. In this case, the presence of Si-containing groups was most likely to be the decisive factor influencing the behavior of cells.

In terms of future studies, for analysing the obtained results to assess the effect of electrical stimulation of the obtained materials on cell adhesion, spreading and, consequently, tissue regeneration, the most desirable samples are CNF_GO and CNF_SiOC.

The obtained results indicate strong potential for the tested samples in contact with bone and cartilage cells, and once again prove that the cellular response depends on a number of physicochemical factors and their mutual synergy.

## Figures and Tables

**Table 1:** Total porosity of CNF before and after modification.

Samples	CNF	CNF_ox	CNF_NaH	CNF_GO	CNF_SiOC
Porosity [%]	98.3	97.9	96.8	97.7	97.1

**Table 2:** Elemental composition of CNFs and modified CNFs obtained from XPS analysis.

Sample	Atomic Concentration [at. %]					
	C	O	N	Si	Na	O/C
CNF	85.8	6.9	7.3	-	-	0.08
CNF_ox	79.0	18.1	2.9		-	0.23
CNF_NaH	68.4	26.4	4.3	-	0.9	0.39
CNF_GO	73.8	25.0	1.2	-	-	0.34
CNF_SiOC	72.6	7.8	1.6	18.0	-	0.11

## Data Availability

The data presented in this study are available on request from the corresponding author. At the time the project was carried out, there was no obligation to make the data publicly available.
